# Association between breastfeeding and prevalence of allergies among children in the Academy Teaching Charity Hospital, Sudan

**DOI:** 10.5339/qmj.2023.sqac.19

**Published:** 2023-11-26

**Authors:** Maab Kamaledlin Mohammedali Mohammed Abouda, Rabih Berair

**Affiliations:** ^1^Pediatrics Department in the University of Medical Sciences and Technology, Sudan Email: MMohammed45@hamad.qa; ^2^Faculty of Pediatrics in Academy Charity Teaching Hospital (ACTH), Sudan

## Abstract

**Background:** The role of breastfeeding in the primary prevention of allergic diseases remains controversial, with hardly any reported studies from developing countries.

**Objective:** To evaluate the association between breastfeeding and the presence of allergies. Specifically, we aimed to demonstrate the association between the exclusivity of breastfeeding and the prevalence of allergies, including asthma, eczema, allergic rhinitis, and food allergy. Secondly, to ascertain the impact of feeding cow milk as complimentary feeding on the prevalence of atopy. Finally, we intended to substantiate the association between maternal education and breastfeeding awareness.

**Materials and Methods:** A cross-sectional study was conducted; 182 participants were enrolled. A confidential, anonymous questionnaire was administered to the participantsf mothers (those attending Academy Charity Teaching Hospital with children aged six months to 10 years). Crude associations between exclusive or non-exclusive breastfeeding and atopic diseases were evaluated using Chi-Square Test by computing crude odds ratios (OR) and corresponding 95% Confidence Interval (CI) with α level of 0.05. Multiple logistic regression models were used to estimate the effect of exclusive breastfeeding on allergic diseases. The sample was adjusted for confounding factors such as gender, family history, number of siblings, and paternal and/or maternal smoking habits.

**Results:** Of the 182 participants included in this study, 95(52.2%) were not exclusively breastfed, and 87(47.8%) were exclusively breastfed. The prevalence of allergic diseases in the children who received non-exclusive breastfeeding compared with those with exclusive breastfeeding showed a significantly higher prevalence of atopy [OR =3.6 95%CI: 1.87-6.94] with a p-value <0.001, even more, significant when the sample was adjusted for family history [OR =4.59 95%CI: 1.96-10.75] with a p-value <0.001. Moreover, milk administration as a complementary feeding type was not significantly associated with the development of atopy [OR =1.65 95%CI: 0.87-3.14] with a p-value of 0.14.

**Conclusion:** There is evidence that exclusive breastfeeding is protective against atopy. Mothers should breastfeed mainly for the first six months of child age and continue with partial breastfeeding beyond that age.

## Figures and Tables

**Figure 1.**
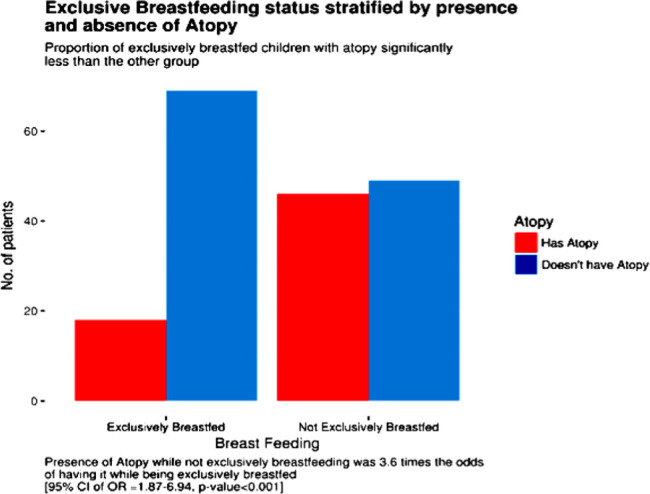
The remarkable difference in the proportion of atopic patients between those who were exclusively breastfed vs. those who were not with an overall [*OR =3.6, 95% CI: 1.87-6.94] and a p-value <0.001*.
